# Increased Lymphatic Vessels: A Risk Factor for Severe Renal Function Loss in Obstructive Nephropathy Patients

**DOI:** 10.7150/ijms.100367

**Published:** 2024-08-26

**Authors:** Zheng Wang, Danni Hu, Huzi Xu, Rui Zeng, Ying Yao

**Affiliations:** 1 Division of Nephrology, Tongji Hospital, Tongji Medical College, Huazhong University of Science and Technology, 1095 Jiefang Avenue, Wuhan, 430030, China.; 2 Key Laboratory of Organ Transplantation, Ministry of Education; NHC Key Laboratory of Organ Transplantation; Key Laboratory of Organ Transplantation, Chinese Academy of Medical Sciences, Wuhan, China.; 3 Department of Nutrition, Tongji Hospital, Tongji Medical College, Huazhong University of Science and Technology, 1095 Jiefang Avenue, Wuhan, 430030, China.

**Keywords:** lymphatic vessels, obstructive nephropathy, risk factor

## Abstract

**Background:** Obstructive nephropathy (ON), resulting from hindered urine flow, significantly contributes to both acute kidney injury (AKI) and chronic kidney disease (CKD). Research has consistently highlighted increased lymphatic vessels (LVs) density in diverse kidney diseases. However, the precise involvement of LVs in ON remains unclear.

**Methods:** Patients diagnosed with ON were enrolled in this study from January 2020 to December 2023. LVs and histological pathology in renal biopsy tissues were detected through immunohistochemistry and Periodic Acid-Schiff staining. Patients were categorized into two cohorts based on their estimated glomerular filtration rate (eGFR) levels: one cohort included patients with eGFR < 90, while the other encompassed those with eGFR ≥ 90. Univariate and multivariable logistic regression analyses were conducted to determine the odds ratio (OR) and 95% confidence interval (CI) for the association between the two cohorts.

**Results:** 239 patients were enrolled in the study. The density of LVs was elevated in ON, with even higher densities observed in patients with severe renal impairment. Additionally, several risk factors contributing to the deterioration of renal function in ON patients have been identified, including age, ureteral calculi (UC), alanine aminotransferase (ALT), and uric acid (UA). Furthermore, by leveraging LVs density, multiple robust models have been established to predict severe renal impairment in ON.

**Conclusions:** Lymphatic vessels density is significantly elevated in ON, serving as an independent risk factor for the decline in renal function.

## Introduction

Obstructive nephropathy (ON), a renal condition stemming from hindered urine flow, can manifest across all age groups, with a myriad of causes, ranging from benign to malignant 1-3. Ureteral obstruction significantly affects kidney function and structure, with the extent of changes and resulting pathological outcomes heavily influenced by the duration, severity, and location of the obstruction.

Acute kidney injury (AKI) is a complex syndrome with widespread effects beyond renal functions. It can impact multiple bodily systems, potentially leading to multiorgan failure and significant biological consequences 4, 5. ON is a common cause of AKI, particularly in acute cases, affecting 5 to 10% of all AKI incidents. In elderly patients, the prevalence of ON can rise to 22% of AKI presentations 6-8. Except that, ON is also a significant factor in chronic kidney disease (CKD) like other factors, such as hypertension, diabetes, and cardiovascular diseases 9, 10. Therefore, further research is still needed on the clinical factors affecting renal function in patients with ON.

The kidney's lymphatic vessels (LVs) system is complex and serves multiple functions, including clearing tissue fluid, cells, and small molecules, and transporting soluble antigens and immune cells between the kidneys and lymph nodes 11, 12. Recent research on LVs, particularly the identification of markers such as LYVE-1, has stimulated interest in comprehending lymphangiogenesis following kidney damage 11, 13. Examination of renal biopsy specimens from patients with various renal pathologies has revealed the emergence of LVs in response to diverse kidney injuries and diseases 14, 15. Notably, an increase in the cross-sectional area of LVs has been observed in renal biopsy samples from patients with conditions like diabetic nephropathy, IgA nephropathy, lupus nephritis, and kidney transplantation 16-20. Nevertheless, the involvement of LVs in ON remains elusive and requires additional investigation.

In this study, our focus was on exploring and screening the risk factors associated with severe renal function impairment in patients diagnosed with ON. Initially, we examined the distribution and expression of LVs in the renal tissues of these patients. Subsequently, we utilized both univariate and multivariate logistic regression analyses to develop a predictive model for renal function impairment, which included variables such as LVs density. This study represents the first identification of LVs density as an independent risk factor for severe renal function impairment in patients with ON, thereby paving the way for further research in this field.

## Methods

### Patients

**Figure 1:**
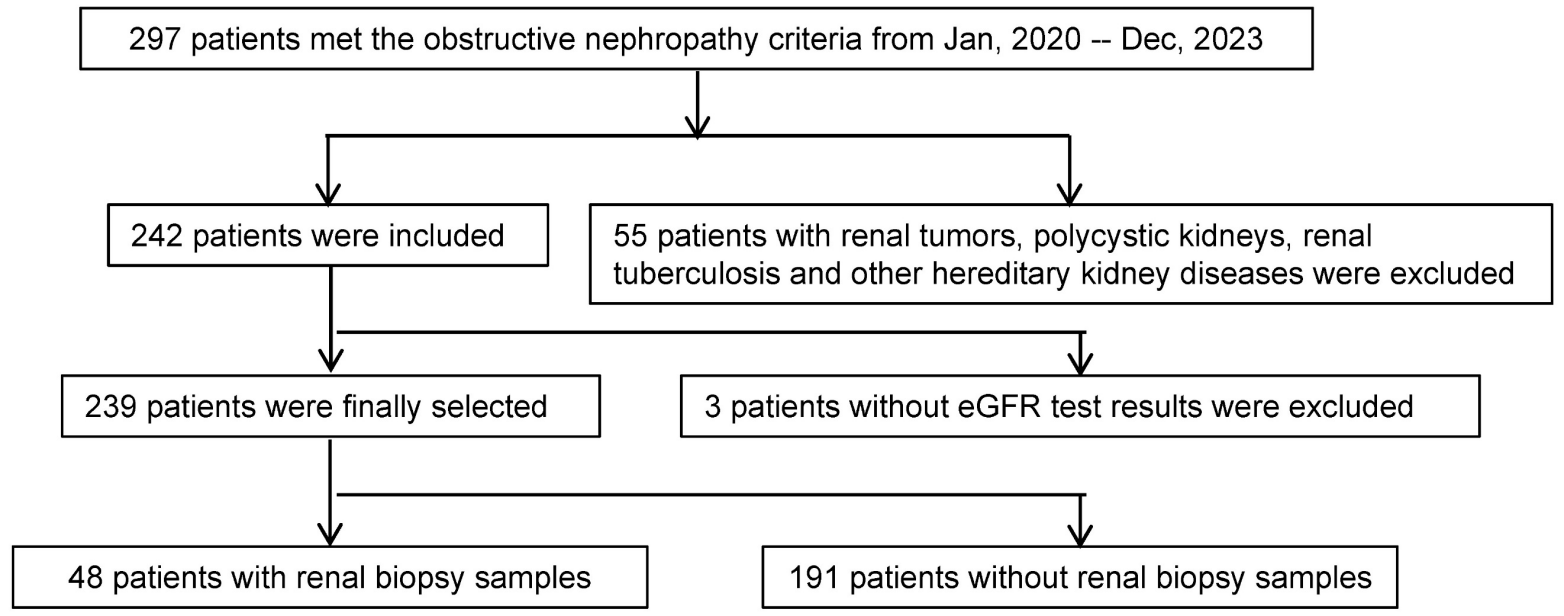
** Study scheme.** The patients were recruited into the study according to the scheme shown. eGFR, Estimated Glomerular Filtration Rate.

This study adhered to the principles outlined in the Helsinki Declaration and received approval from the Research Ethics Committee of Tongji Hospital, Huazhong University of Science and Technology. Over the period spanning January 2020 to December 2023, a total of 297 patients received diagnoses of obstructive nephropathy. Out of this cohort, 55 patients were excluded due to conditions such as renal tumors, polycystic kidneys, renal tuberculosis, and other genetic kidney diseases. Additionally, an additional 3 patients were excluded due to the absence of estimated glomerular filtration rate (eGFR) results. Ultimately, among the initially screened 239 patients, 48 had renal biopsy samples (Figure 1).

### Clinical and laboratory data

The following information was collected in the present study: gender, age, blood pressure, body mass index (BMI), hematuria, proteinuria, uric acid (UA), serum bicarbonate (HCO3) levels, serum creatinine (Scr), eGFR, as well as other results from blood tests, urine analysis, and imaging examinations. According to imaging examinations, we categorize ureteral calculi (UC) into upper, middle, and lower UC.

### Histological evaluation

Paraffin sections were stained with Periodic Acid-Schiff and Masson's trichrome for pathological evaluation. Pathological features were assessed, including evaluations of immune cell infiltration and tubular atrophy/interstitial fibrosis. In order to minimize errors due to variations in sectioning levels, successive slices of the renal biopsy specimen were placed on a unified slide. Following the staining and procedural stages, two independent pathologists examined these sequential sections.

### Immunohistochemistry

Immunohistochemical staining was conducted on kidney tissue paraffin sections using the D2-40 antibody (Abcam, Cambridge, UK) to detect LVs in renal biopsies 21. An immunohistochemistry kit from Dako (Santa Clara, CA, USA) was utilized for this purpose. Visualization was achieved through the application of 3,3ʹ-diaminobenzidine (DAB). Co-localization studies were performed on consecutive sections. The ten regions with the highest lymphatic vessel density were identified as hotspots, and all counting assessments were independently carried out by two experienced pathologists.

### Statistical analyses

Statistical analyses were performed using IBM SPSS Statistics version 26.0 (IBM Corp., Armonk, NY, USA) and R version 4.2.2. Variables were presented as median with interquartile range (IQR) or proportion, as appropriate. Nonparametric statistics were employed to evaluate between-group variances and associations. Correlations were assessed using Spearman's rank correlation coefficient for continuous variables, and the Mann-Whitney U test was used for median comparisons. Rate comparisons were performed using chi-squared tests. Univariate and multivariable logistic regression analyses were conducted to assess the odds ratio (OR) and 95% confidence interval (CI) for the association between ON patients categorized by eGFR (≥ 90 vs. < 90). All statistical tests were two-tailed.

## Results

### Increased lymphatic vessel density in obstructive nephropathy correlates closely with kidney damage and impaired renal function

An increasing amount of research suggests LVs are important in kidney diseases. Our team has found the elevated LVs in kidney diseases like IgA nephropathy and crescentic glomerulonephritis 17, 22. However, there is little research on ON. Therefore, this study aims to investigate the effect of LVs on ON.

**Figure 2:**
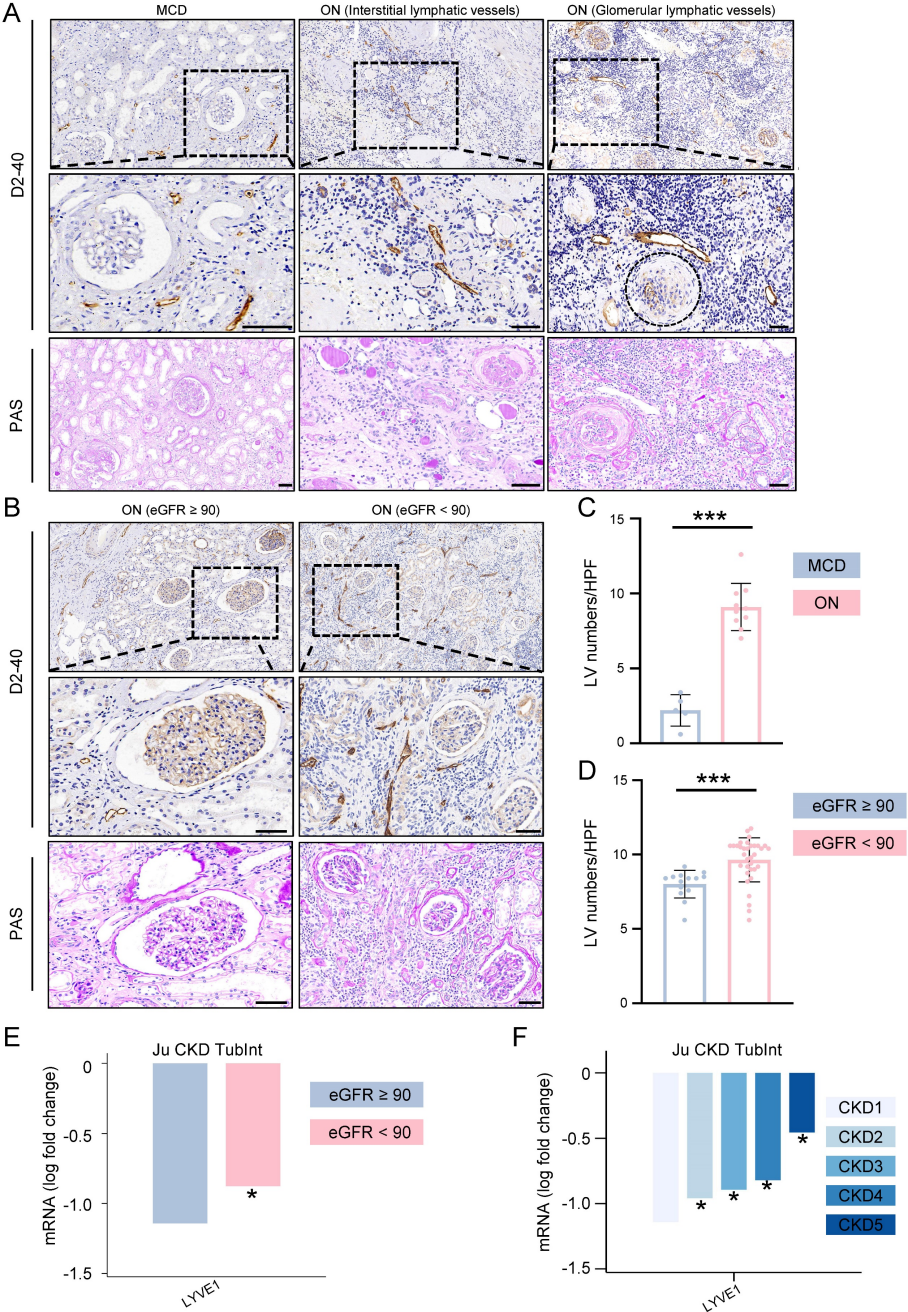
** The density of lymphatic vessles was incresed in obstructive nephropathy.** A. Obstructive nephropathy patients' renal biopsy specimens were stained using PAS for histopathological analysis, and D2-40 for immunohistochemical evaluation on consecutive sections. The glomerulus is localized within the demarcated regions delineated by the black dashed line. B. Obstructive nephropathy patients' (eGFR ≥ 90 and eGFR < 90) renal biopsy specimens were stained using PAS for histopathological analysis, and D2-40 for immunohistochemical evaluation on consecutive sections. C. Bar plot illustrating the lymphatic vessel counts in patients with MCD and ON. D. Bar plot illustrating the lymphatic vessel counts in obstructive nephropathy patients across two groups stratified by eGFR values (eGFR ≥ 90 and eGFR < 90). E. Bar plot illustrating the expression levels of LYVE1 in patients stratified by eGFR values (eGFR ≥ 90 and eGFR < 90). F. Bar plot illustrating the expression levels of LYVE1 in patients with CKD across different stages (CKD1, CKD2, CKD3, CKD4, and CKD5). MCD, Minimal Change Disease; ON, Obstructive Nephropathy; eGFR, Estimated Glomerular Filtration Rate; CKD, Chronic Kidney Disease; HPF, High-power field; *** p <0.001; *p <0.05, Scale bar: 50 μm.

ON, in comparison to minimal change disease (MCD), demonstrates a pronounced elevation in LVs density throughout the glomeruli and interstitium (Figure 2A and 2C). The regions abundant in LVs also experience a substantial influx of immune cells, further aggravating renal injury, encompassing both tubular and glomerular damage.

Based on the eGFR levels of ON patients, we categorized them into two groups: eGFR ≥ 90 and eGFR < 90. Our findings revealed that the eGFR < 90 group exhibited a higher density of LVs within the kidneys when contrasted with the eGFR ≥ 90 group. Additionally, this group displayed notable immune cell infiltration and kidney tissue damage (Figure 2B and 2D).

Next, we investigated the expression of LYVE1, the gene symbol for LVs, by analyzing the Nephroseq database (https://www.nephroseq.org). In the 'Ju CKD Tublnt' dataset, LYVE1 mRNA exhibited significant upregulation in kidney biopsy tissues of patients with eGFR < 90 compared to those with eGFR ≥ 90. Moreover, the expression level of LYVE1 increased progressively with declining kidney function (Figure 2E and 2F).

### Baseline clinical characteristics of all enrolled patients

A total of 239 patients with ON were included in the study. Based on the patients' eGFR levels, they were divided into two groups: eGFR ≥ 90 group (n = 51) and eGFR < 90 group (n = 188) (Table 1). The median age of the patients was 55 years (47.5 - 62.5), with male patients accounting for 46.44% (n = 111). The median systolic blood pressure of the patients was 130 mmHg (120 - 136), with no significant difference between the two groups. The median BMI index of the patients was 23.4 kg/m² (21.8 - 26.0), higher in the eGFR < 90 group. The proportion of patients with UC was 57.32% (n = 137), significantly higher in the eGFR < 90 group, but there was no statistically significant difference in the distribution of UC locations between the two groups. Furthermore, differences were observed in red blood cells, albumin (ALB), Albumin/Globulin (A/G) ratio, ALT, Triglyceride (TG), High-density lipoprotein (HDL), Cl-, and Na+ between the two groups. Indicators closely related to renal function, such as Cystatin C (CysC), Scr, and UA, also showed significant differences between the two groups.

### Identifying the risk factors associated with the severity of kidney function loss in obstructive nephropathy

To delve deeper into factors closely linked to severe kidney function loss, we conducted both univariate and multivariate logistic regression analyses. Initially, we included variables that exhibited disparities between the aforementioned two groups. These variables encompassed gender, age, BMI, UC, red blood cells, ALB, A/G ratio, ALT, Cl-, Na+, and UA. Furthermore, we integrated indicators such as nephrolithiasis and HCO3 levels. However, TG and HDL were omitted from the analysis due to a substantial number of missing values. Additionally, Scr and CysC were excluded owing to their linear correlation with eGFR.

After conducting logistic regression analysis, we identified that age (OR = 1.08, 95% CI: 1.04-1.13, p < 0.001), UC (OR = 3.11, 95% CI: 1.29-7.49, p = 0.012), ALT (OR = 1.07, 95% CI: 1.01-1.13, p = 0.018), and UA (OR = 1.02, 95% CI: 1.01-1.02, p < 0.001) serve as independent risk factors for severe loss in kidney function (Table 2).

### Increased lymphatic vessel density is an independent risk factor for the deterioration of renal function in patients with obstructive nephropathy

Previous results indicate an increase in LVs density among ON patients, with a notably higher density observed in the eGFR < 90 group compared to those with eGFR ≥ 90. D2-40 staining was then conducted on renal pathology specimens from patients to quantify LVs density. Patients were stratified into two cohorts according to eGFR levels, followed by comprehensive descriptive and statistical analyses of both the previously identified variables and LVs density.

A total of 48 patients were included in the analysis and divided into two groups based on their eGFR levels: eGFR ≥ 90 (n=14) and eGFR < 90 (n=34) groups. The median age of the patients was 53 years (41 - 58), with males accounting for 47.92% (n=23). There was no significant difference in systolic blood pressure between the two groups, with a median of 125 mmHg (119-131). The BMI index of the patients was 23.3 kg/m² (22.4 - 26.7), higher in the eGFR < 90 group. The prevalence of UC was 47.91% (n=23), notably increased in the eGFR < 90 group. Additionally, differences were observed between the two groups in parameters such as TG, HDL, CysC, Scr, and UA. LVs density also showed a significant increase in the eGFR < 90 group (Table 3). Similar trends were observed in both the overall and subgroup analyses.

**Figure 3:**
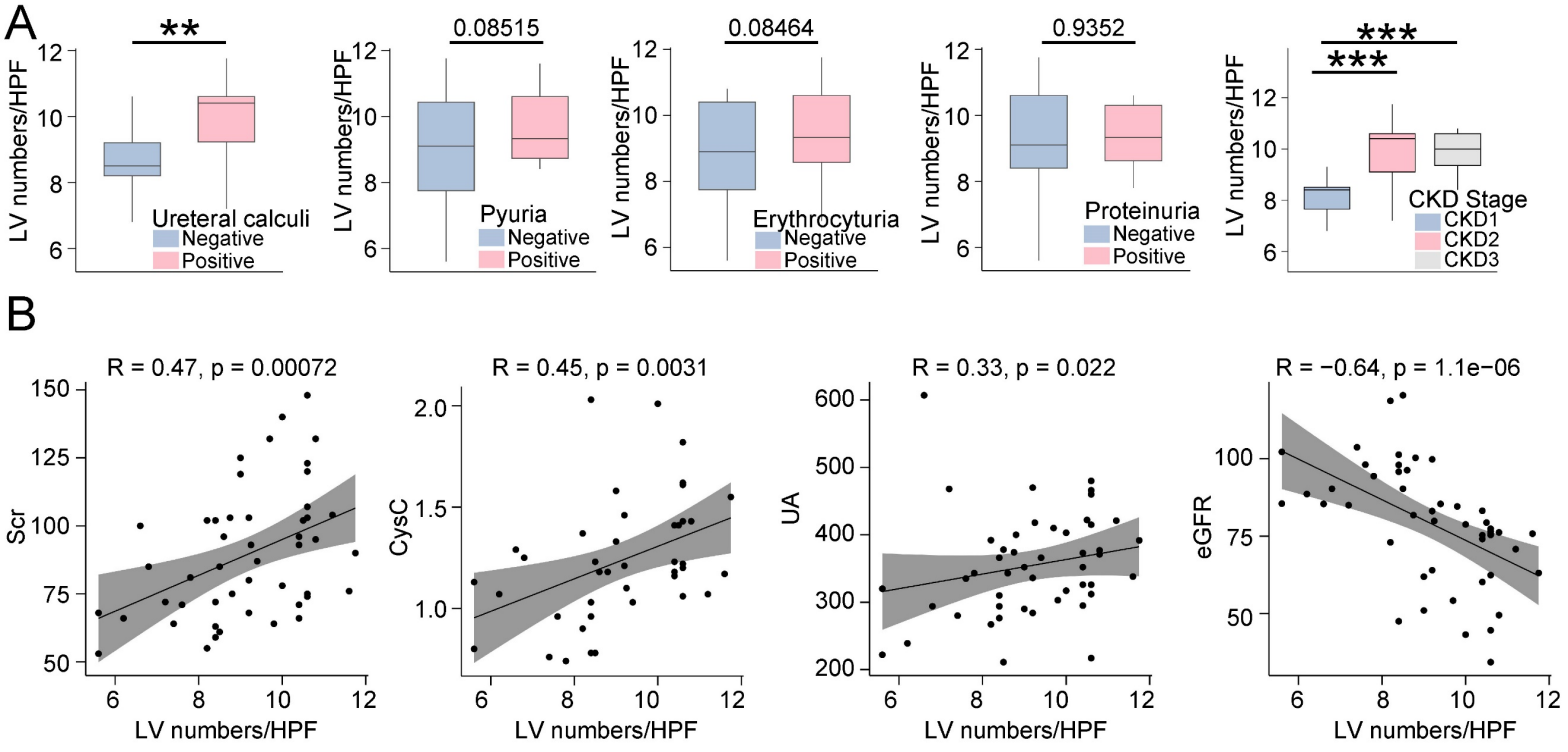
** The density of lymphatic vessles was correlated with various clinic features among patients with obstructive nephropathy.** A. Box plots illustrating the lymphatic vessel counts in obstructive nephropathy patients categorized by presence of ureteral calculi (positive, negative), pyuria (positive, negative), hematuria (positive, negative), proteinuria (positive, negative), and CKD stages. B. Scatter plots illustrating the correlation between lymphatic vessel counts in renal biopsy specimens and the levels of Scr, CysC, UA, and eGFR in patients diagnosed with obstructive nephropathy, utilizing Spearman's rank correlation analysis. CysC, Cystatin C; Scr, Serum creatinine; UA, Uric acide; GFR, Estimated Glomerular Filtration Rate; HPF, High-power field; CKD, Chronic kidney disease; **p<0.01, ***p <0.001.

Upon further investigation into the relationship between LVs and other clinical variables, we observed that LVs density was significantly higher in patients with UC compared to those without. Additionally, in patients positive for pyuria, hematuria, and proteinuria, LVs density was higher compared to those without, although there was no statistical difference between the two groups. Our findings also revealed a correlation between LVs and CKD stages in patients with ON, indicating an increase in LVs with CKD progression (Figure 3A). Furthermore, LVs density showed a positive correlation with Scr, CysC, and UA levels but a negative correlation with eGFR levels (Figure 3B).

Pathological injuries such as tubular damage, interstitial fibrosis, and global glomerulosclerosis are closely correlated with the prognosis of kidney diseases. Therefore, we conducted a statistical analysis of these pathological features in patients diagnosed with ON and explored their correlation with LVs (Figure S1A and S1B). Patients were categorized into LVs^low^ and LVs^high^ groups based on the average LVs density. Among ON patients, the median area of interstitial fibrosis was 30.1 (25.4-32.2), significantly higher in the LVs^high^ group. The median score for tubular damage was 3.3 (3.1-3.7), also notably elevated in the high-density LVs group. Regarding global glomerulosclerosis, the median was 30.4 (27.6-31.5), remaining significantly elevated in the high-density LVs group (Table S1). Further analysis revealed a positive correlation between LVs density and both interstitial fibrosis and tubular damage. However, the correlation between LVs density and global glomerulosclerosis did not achieve statistical significance (Figure S1C).

In order to explore the association between LVs density and severe impairment of renal function, our study involved conducting univariate and multivariate logistic regression analyses. Initially, we integrated the independent risk factors identified in prior comprehensive analyses into the model, which comprised age, UC, ALT, and UA. Furthermore, we expanded the analysis to encompass BMI and gender.

**Figure 4:**
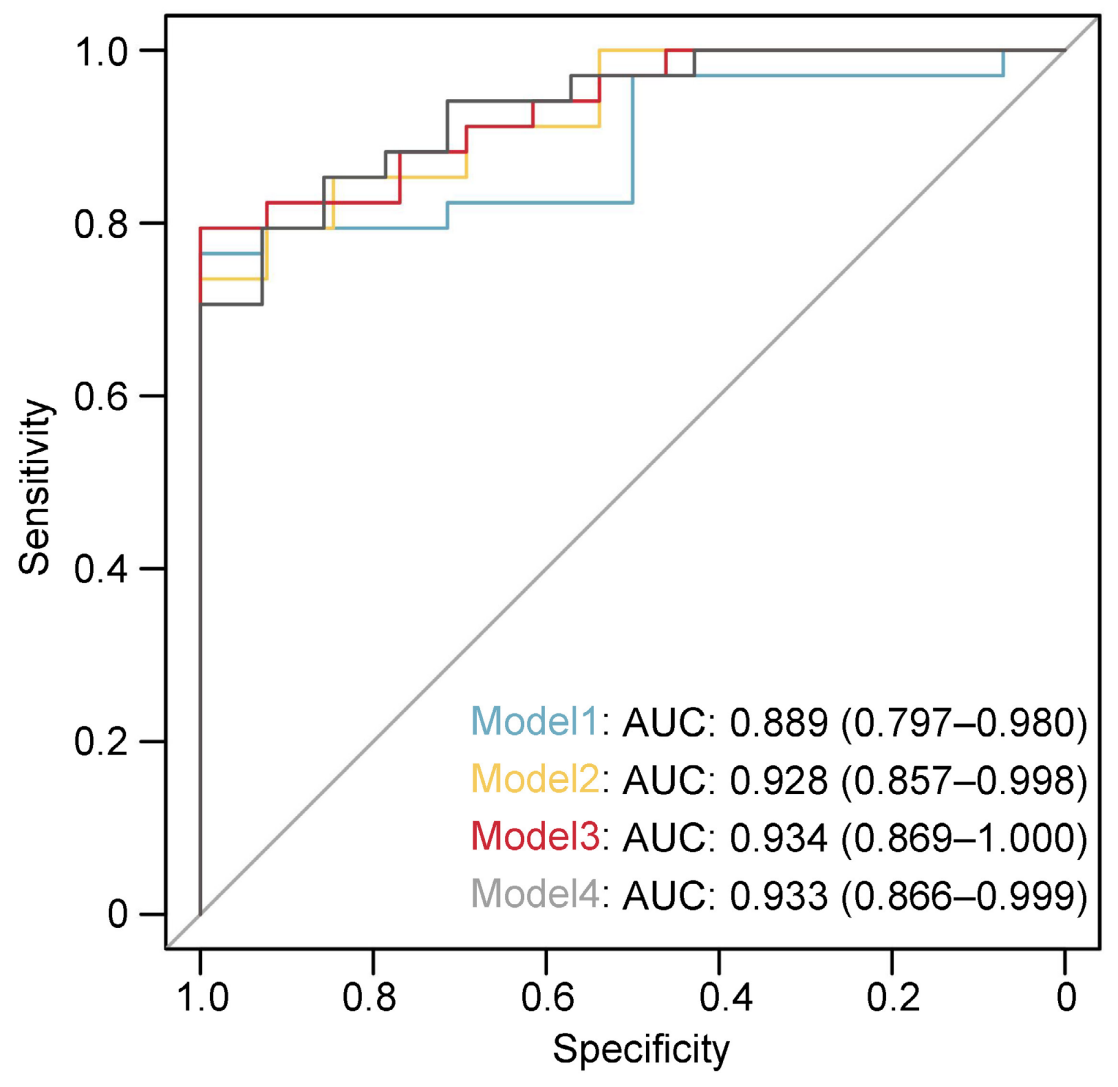
** Receiver operating characteristics (ROC) curve for the discriminative effect of the LVs-based models.** AUC, the area under the curve.

In the univariate logistic regression analysis, we found the following factors to be statistically significant: age (OR=1.80, 95% CI: 0.49 - 6.64, p=0.006), BMI (OR=1.46, 95% CI: 1.11 - 1.92, p=0.004), UC (OR=8.8, 95% CI: 1.69 - 46.63, p=0.01), UA (OR=1.02, 95% CI: 1.00 - 1.03, p=0.014), and LVs density (OR=2.25, 95% CI: 1.30 - 3.89, p=0.04). Subsequently, multiple multivariate logistic regression models were executed to adjust for the LVs density, consistently illustrating the superior efficacy of LVs density. Specifically, Model 1 (including age and gender) showed an OR of 2.19 with a 95% CI of 1.22 - 3.92 and a p-value of 0.008. In Model 2 (encompassing gender, age, and BMI), the OR was 2.47 with a 95% CI of 1.26 - 4.82 and a p-value of 0.008. Furthermore, Model 3 (integrating gender, age, BMI, and UC) presented an OR of 2.37 with a 95% CI of 1.18 - 4.74 and a p-value of 0.015. Lastly, Model 4 (involving gender, age, and UA) exhibited an OR of 2.21 with a 95% CI of 1.12 - 4.3 and a p-value of 0.023 (Table 4). Additionally, we computed the area under the receiver operating characteristic curve (AUC-ROC) for each model, yielding values of 0.889 for Model 1, 0.928 for Model 2, 0.933 for Model 3, and 0.934 for Model 4 (Figure 4). These AUC-ROC values demonstrate the high diagnostic efficiency of the models.

## Discussion

Our research has revealed that LVs density escalates in ON, with even higher densities observed in individuals with severe renal impairment. Additionally, we have identified risk factors contributing to the deterioration of renal function in ON patients, which include age, UC, ALT, and UA. Moreover, leveraging LVs density, we have formulated multiple robust models for predicting severe renal impairment. These discoveries furnish clinical directives for forthcoming diagnostic and therapeutic strategies in ON, while also introducing novel perspectives and methodologies for delving deeper into the role of LVs in this condition.

The discovery of LVs-specific markers, such as LYVE-1 and VEGFR3, has expedited research into the involvement of LVs in diseases 23-26. Recent studies have demonstrated an augmented expression of LVs in various kidney diseases, including CKD, IgA nephropathy, lupus nephritis, and kidney transplant rejection, underscoring the increasing significance of LVs in renal pathologies 16-20. Our study has similarly revealed heightened expression levels of LVs in ON, exhibiting a widespread distribution surrounding the renal glomeruli and tubulointerstitium. This further underscores the pivotal role of LVs in renal diseases.

Proliferation of LVs, termed lymphangiogenesis, remains contentious regarding its precise implications in kidney diseases 14. The formation of LVs plays a crucial role in the elimination of inflammatory cells within damaged tissue, thereby playing a pivotal role in resolving inflammation and preventing fibrosis. Blocking the function of renal lymphatic vessels triggers a cascade of undesired effects, including the deterioration of renal function, the development of tubulointerstitial fibrosis, and the proliferation of interstitial cells 27, 28. Nevertheless, in areas with increased LVs density, a surge in immune cell infiltration is observed, encompassing dendritic cells, T cells, and B cells, which coincides with the acceleration of tubulointerstitial fibrosis 16, 17, 29. In our study, we noted elevated LVs density in cases where the eGFR fell below 90 mL/min/1.73m2. Elevated LVs density in specific regions correlated with an augmented presence of inflammatory cells, exacerbating injury to renal tubules and glomeruli. Furthermore, we observed a positive association between LVs density and levels of Scr, CysC, and UA, while noting a negative correlation with eGFR. The upregulation of the LVs marker gene LYVE1 corresponded with the deterioration of renal function in patients with CKD. Our developed predictive model for renal dysfunction, integrating LVs density, exhibited robust predictive capabilities and identified LVs density as an independent risk predictor. These findings indicate that elevated LVs density might contribute to the aggravation of renal injury in ON. Nonetheless, elucidating the precise mechanisms underlying the involvement of LVs in this process necessitates further inquiry, laying a foundational framework for investigating the role of lymphatic vessels in ON.

ON is a crucial factor in AKI and CKD. Understanding its risk factors is crucial for diagnosis, treatment, and prevention. Besides dietary factors like obesity, diabetes, gout, and hypertension, non-dietary factors also play a significant role. These factors can lead to urinary system stones by affecting the excretion of calcium and uric acid in urine and altering urine pH 3, 30-33. Additionally, stone size and location are further risk factors for ON and renal function impairment. In our study, differences were observed in age, BMI, UC, red blood cell, ALB, ALT, TG, HDL, Cl-, Na+, and UA between the eGFR < 90 and eGFR ≥ 90 groups. Additionally, age, UC, ALT, and UA were identified as independent risk factors associated with impaired renal function in ON. These findings may provide valuable insights for guiding clinical diagnosis and treatment.

This study has a number of limitations. The sample size of pathological samples is limited, necessitating an increase in sample size to enhance the credibility of the research. When evaluating the factors contributing to ON, only common urinary system factors were taken into account, while external factors such as abdominal tumors causing obstruction were not considered. Limitations in imaging results hindered a thorough examination of stone characteristics, such as size and shape. The study reveals that the density of LVs constitutes an independent risk factor for severe kidney damage in ON, but the exact mechanism by which they operate remains unclear, emphasizing the requirement for further research to clarify this aspect.

## Supplementary Material

Supplementary figure and table.

## Figures and Tables

**Table 1:** Baseline characteristics of all enrolled patients.

Characteristic	Overall, N=239	eGFR ≥ 90, N=51	eGFR < 90, N=188	p-value*^1^*
Gender male, n (%)	111 (46.44)	17 (33.33)	94 (50.0)	**0.034**
Age, years	55 (47.5 - 62.5)	48 (36 - 56)	56 (50 - 66)	**< 0.001**
SBP	130 (120 - 136)	125 (119.5 - 131.5)	130 (120 - 138)	0.073
DBP	80 (75 - 86.5)	80 (75 - 84)	80 (76.75 - 87)	0.28
BMI	23.4 (21.8 - 26.0)	22.9 (19.8 - 24.8)	23.8 (22.3 - 26.1)	**0.005**
UC positive, n (%)	137 (57.32)	18 (35.29)	119 (63.30)	**< 0.001**
UC position, n (%)				0.94
Upper	76 (57.14)	11 (57.90)	65 (57.02)	
Middle	25 (18.80)	4 (21.05)	21 (18.42)	
Lower	32 (24.06)	4 (21.05)	28 (24.56)	
Nephrolithiasis, n (%)	112 (46.86)	22 (43.14)	90 (47.87)	0.55
IUS positive, n (%)	50 (20.92)	22 (43.14)	90 (47.87)	0.93
Pyuria positive, n (%)	100 (42.55)	19 (38)	81(43.78)	0.46
Erythrocyturia, n (%)	95 (40.42)	19 (38)	76 (41.08)	0.69
Proteinuria positive, n (%)	80 (34.04)	12 (24)	68 (36.76)	0.091
Crystalluria, n (%)	14 (9.46)	4 (12.12)	10 (8.70)	0.51
WBC	5.56 (4.64 - 6.88)	5.56 (4.76 - 5.94)	5.56 (4.58 - 6.99)	0.40
Mono	0.43 (0.35 - 0.55)	0.41 (0.33 - 0.51)	0.44 (0.36 - 0.56)	0.10
RBC	4.30 (4.00 - 4.63)	4.48 (4.13 - 4.73)	4.28 (3.96 - 4.58)	**0.022**
Lymph	1.73 (1.30 - 2.10)	1.77 (1.31 - 2.20)	1.71 (1.30 - 2.08)	0.50
Baso	0.02 (0.01 - 0.03)	0.02 (0.01 - 0.03)	0.02 (0.01 - 0.04)	0.43
Eosi	0.14 (0.08 - 0.21)	0.13 (0.08 - 0.21)	0.14 (0.09 - 0.21)	0.68
HGB	128 (118 - 139)	130 (120.5 - 139)	128 (117 - 138.25)	0.62
PLT	218 (181.5 - 260)	219 (183 - 255.5)	217 (180.75 - 260)	0.95
Neutro	3.19 (2.51 - 4.08)	3.16 (2.57 - 3.57)	3.22 (2.50 - 4.20)	0.37
ALB	40.4 (38.5 - 42.4)	41 (40 - 42.65)	40.3 (38.18 - 42.4)	**0.03**
GLB	28.6 (25.85 - 31.05)	28.2 (25.95 - 30.15)	29.05 (25.78 - 31.65)	0.17
A/G	1.43 (1.26 - 1.62)	1.49 (1.38 - 1.64)	1.4 (1.25 - 1.59)	**0.038**
TP	69.2 (65.9 - 71.95)	69.4 (67.7 - 71.3)	69.15 (65.78 - 72.03)	0.79
AST	13 (10 - 20)	12 (9.5 - 18)	13.5 (10 - 20)	0.15
ALT	17 (15 - 21)	16 (14.5 - 18)	17 (15 - 21)	**0.026**
DB	3.4 (2.7 - 4.3)	3.5 (2.9 - 4.55)	3.3 (2.6 - 4.3)	0.48
IB	5.6 (3.93 - 7.6)	5.6 (4.9 - 7.7)	5.6 (3.75 - 7.6)	0.40
TB	8.9 (6.8 - 11.75)	9.2 (7.65 - 11.65)	8.75 (6.48 - 11.83)	0.30
TG	1.23 (0.91 - 1.58)	0.98 (0.71 - 1.25)	1.36 (1.04 - 1.89)	**< 0.001**
HDL	1.11 (0.93 - 1.32)	1.22 (1.11 - 1.48)	1.05 (0.88 - 1.29)	**0.002**
CHOL	4.14 (3.63 - 4.71)	4.35 (3.78 - 4.78)	4.12 (3.57 - 4.70)	0.31
LDL	2.66 (2.15 - 3.13)	2.70 (2.18 - 3.24)	2.65 (2.16 - 3.04)	0.58
K^+^	4.12 (3.92 - 4.31)	4.09 (3.95 - 4.23)	4.13 (3.91 - 4.32)	0.35
Ca^2+^	2.27 (2.22 - 2.34)	2.27 (2.23 - 2.32)	2.27 (2.22 - 2.35)	0.6
P	1.10 (0.94 - 1.23)	1.15 (0.98 - 1.24)	1.09 (0.93 - 1.22)	0.28
Cl^-^	104.7(103.15 - 106.1)	104.1(102.75 - 105.3)	104.95(103.3 - 106.3)	**0.019**
Mg^2+^	0.84 (0.8 - 0.87)	0.83 (0.8 - 0.86)	0.85 (0.8 - 0.89)	0.12
Na^+^	140.55 (139.2 - 141.9)	139.9(138.6 - 140.95)	140.6 (139.45 - 142)	**0.024**
CysC	1.21 (1.07 - 1.43)	1.03 (0.9 - 1.18)	1.35 (1.17 - 1.56)	**<0.001**
Scr	89 (72.5 - 109.5)	65 (60.5 - 75)	97 (79 - 115)	**<0.001**
UA	358(301 - 418.5)	297(256.75 - 339.5)	371.75(318.5 -433.78)	**<0.001**
HCO3	23.1 (21.45 - 24.9)	24.10 (22.2 - 25.05)	22.9 (21.3 - 24.8)	0.072
Glu	5.06 (4.70 - 5.52)	5.08 (4.69 - 5.40)	5.06 (4.70 - 5.61)	0.77

The bold values indicate P < 0.05. Data are presented as median (25-75th percentiles) or a percentage. ***^1^*** Pearson's Chi-squared test; Wilcoxon rank sum test; Fisher's exact test. SBP, Systolic blood pressure; DBP, Diastolic blood pressure; BMI, Body mass index; UC, Ureteral calculi; Upper, Upper ureteral calculi; Middle, Middle ureteral calculi; Lower, Lower ureteral calculi; IUS, Idiopathic ureteral stenosis; WBC, White blood cell; Mono, Monocyte; RBC, Red blood cell; Lymph, Lymphocyte; Baso, Basophil; Eosi, Eosinophil; HGB, Hemoglobin; PLT, Platelet; Neutro, Neutrophil; ALB, Albumin; GLB, Globulin; A/G, Albumin/Globulin; TP, Total protein; AST, Aspartate aminotransferase; ALT, Alanine aminotransferase; DB, Direct bilirubin; IB, Indirect bilirubin; TB, Total bilirubin; TG, Triglyceride; HDL, High-density lipoprotein; CHOL, Cholesterol; LDL, Low-density lipoprotein; CysC, Cystatin C; Scr, Serum creatinine; UA, Uric acid; Glu, Glucose; eGFR, Estimated glomerular filtration rate.

**Table 2:** Risk factors associated with eGFR < 90 ml/min/1.73 m^2^ in the enrolled patients.

Variables	Univariate analysis			Multivariate analysis		
	OR	95%CI	P	OR	95%CI	P
Gender male	1.90	0.99 - 3.68	0.055	1.19	0.42 - 3.39	0.743
Age, yearrs	1.08	1.05 - 1.11	**< 0.001**	1.08	1.04 - 1.13	**< 0.001**
UC positive	3.35	1.72 - 6.50	**< 0.001**	3.11	1.29 - 7.49	**0.012**
ALT	1.05	0.99 - 1.11	0.113	1.07	1.01 - 1.13	**0.018**
UA	1.01	1.01 - 1.02	**0.001**	1.02	1.01 - 1.02	**< 0.001**

Univariate and multivariate logistic regression analyses of clinical features of enrolled patients. The bold values indicate P < 0.05. OR, Odds ratio; CI, Confidence interval; UC, Ureteral calculi; ALT, Alanine aminotransferase; UA, Uric acid.

**Table 3:** Screened baseline characteristics of enrolled patients with renal biopsy samples.

Characteristic	Overall, N=48	eGFR ≥ 90, N=14	eGFR < 90, N=34	p-value*^1^*
Gender male, n (%)	23 (47.92)	17 (35.71)	18 (52.94)	0.28
Age, years	53 (41 - 58)	44 (34 - 52)	55 (48 - 62)	**0.005**
SBP	125 (119 - 131)	126 (117 - 130)	124 (119 - 133)	0.66
DBP	80 (75 - 85)	78 (74 - 80)	80 (78 - 88)	0.18
BMI	23.3 (22.4 - 26.7)	22.6 (19.6 - 23.1)	25.7 (22.8 - 27.6)	**0.004**
UC positive, n (%)	23 (47.91)	2 (14.29)	21 (61.76)	**0.003**
RBC	4.43 (4.15 - 4.70)	4.37 (4.19 - 4.58)	4.43 (4.14 - 4.81)	0.63
ALB	40.1 (39.3 - 42.48)	41.25 (40.15 - 41.75)	40.95 (39.23 - 42.78)	0.77
A/G	1.47 (1.29 - 1.59)	1.54 (1.40 - 1.65)	1.42 (1.27 - 1.57)	0.18
ALT	16 (15 - 21)	15 (15 - 16.8)	16.5 (15 - 21)	0.22
TG	1.13 (0.88 - 1.45)	0.75 (0.55 - 0.9)	1.38 (1.11 - 1.61)	**< 0.001**
HDL	1.11 (0.99 - 1.29)	1.19 (1.12 - 1.58)	1.05 (0.95 - 1.14)	**0.04**
Cl^-^	104.1 (103.03 - 105.3)	104.65 (103 - 105.08)	103.95 (103.13 - 105.38)	0.89
Na^+^	140.6 (139.6 - 141.78)	140.1 (139.3 - 141.3)	140.6 (139.93 - 142.4)	0.25
CysC	1.2 (1.06 - 1.41)	0.96 (0.79 - 1.18)	1.33 (1.17 - 1.51)	**<0.001**
Scr	86 (71 - 103)	70 (62 - 80)	98 (77 - 106)	**<0.001**
UA	348 (301 - 401)	303 (277 - 341)	372 (322 - 417)	**0.004**
HCO3	23.55 (21.8 - 24.83)	24.35 (22.7 - 25.08)	22.35 (21.8 - 24.78)	0.25
LVs	9.2 (8.4 - 10.53)	8.4 (7.65 - 8.5)	10.2 (9.05 - 10.6)	**<0.001**

The bold values indicate P < 0.05. Data are presented as median (25-75th percentiles) or a percentage. ***^1^*** Pearson's Chi-squared test; Wilcoxon rank sum test; Fisher's exact test. SBP, Systolic blood pressure; DBP, Diastolic blood pressure; BMI, Body mass index; UC, Ureteral calculi; RBC, Red blood cell; ALB, Albumin; A/G, Albumin/Globulin; ALT, Alanine aminotransferase; TG, Triglyceride; HDL, High-density lipoprotein; CysC, Cystatin C; Scr, Serum creatinine; UA, Uric acid; Glu, Glucose; eGFR, Estimated glomerular filtration rate.

**Table 4:** Risk factors associated with eGFR < 90 ml/min/1.73 m2 in the enrolled patients with renal biopsy samples.

Variables	OR	95% CI	P Value
Univariate analysis			
Gender male	1.80	0.49 - 6.64	0.377
Age, years	1.09	1.03 - 1.17	**0.006**
BMI	1.46	1.11 - 1.92	**0.004**
UC positive	8.88	1.69 - 46.63	**0.01**
ALT	1.09	0.93 - 1.28	0.274
UA	1.02	1.00 - 1.03	**0.014**
LVs	2.25	1.30 - 3.89	**0.04**
Multivariate analyses			
LVs in Model1	2.19	1.22 - 3.92	**0.008**
LVs in Model2	2.47	1.26 - 4.82	**0.008**
LVs in Model3	2.37	1.18 - 4.74	**0.015**
LVs in Model4	2.21	1.12 - 4.3	**0.023**

Univariate and multivariate logistic regression analyses of clinical features in renal biopsy samples of ON patients. The bold values indicate P < 0.05. Model1 Adjust for gender and age. Model2 Adjust for gender, age, and BMI. Model3 Adjust for gender, age, BMI, and UC positive. Model4 Adjust for gender, age, and UA. OR, Odds ratio; CI, Confidence interval; UC, Ureteral calculi; ALT, Alanine aminotransferase; UA, Uric acid; LVs, Lymphatic vessels. ON, Obstructive nephropathy.

## Abbreviations

ON: obstructive nephropathy
AKI: acute kidney injury
CKD: chronic kidney disease
LVs: lymphatic vessels
eGFR: estimated glomerular filtration rate
OR: odds ratio
CI: confidence interval
UC: ureteral calculi
ALT: alanine aminotransferase
UA: uric acid
BMI: body mass index
HCO3: serum bicarbonate
Scr: serum creatinine
IQR: interquartile range
MCD: minimal change disease
ALB: albumin
A/G: albumin/globulin
TG: triglyceride
HDL: high-density lipoprotein
CysC: Cystatin C
