# Supplementary material for: Increased Lymphatic Vessels: A Risk Factor for Severe Renal Function Loss in Obstructive Nephropathy Patients
Source: Int J Med Sci. 2024 Aug 26;21(12):2305–14. doi: 10.7150/ijms.100367 (PMC11413892; doi:10.7150/ijms.100367)
Supplement: Supplementary file 1 — Supplementary figure and table. [file ijmsv21p2305s1.pdf]

Supplementary legend

Supplemental.Figure 1. The correlation between lymphatic vessels and histopathological features.

A. Representative micrographs and corresponding interstitial fibrosis area statistical analysis of Masson-trichrome staining in patients with ON and MCD.

B. Representative micrographs and corresponding global glomerulosclerosis proportion statistical analysis of PAS staining in patients with ON and MCD.

C. Scatter plots illustrating the correlation between lymphatic vessel counts in renal biopsy specimens and tubular damage scores, interstitial fibrosis area, and global glomerulosclerosis proportion in patients diagnosed with ON, utilizing Spearman's rank correlation analysis.

MCD, Minimal Change Disease; ON, Obstructive Nephropathy; HPF, High-power field; LV, lymphatic vessel. *** $p < 0.001$; Scale bar: 50 μm.

Supplemental.Table 1. Histopathological features of enrolled patients with renal biopsy samples.

The bold values indicate $P < 0.05$. Data are presented as median (25–75th percentiles). [1] Wilcoxon rank sum test. Based on the average lymphatic vessel density, the samples were categorized into high-density and low-density groups. LVs, lymphatic vessels.

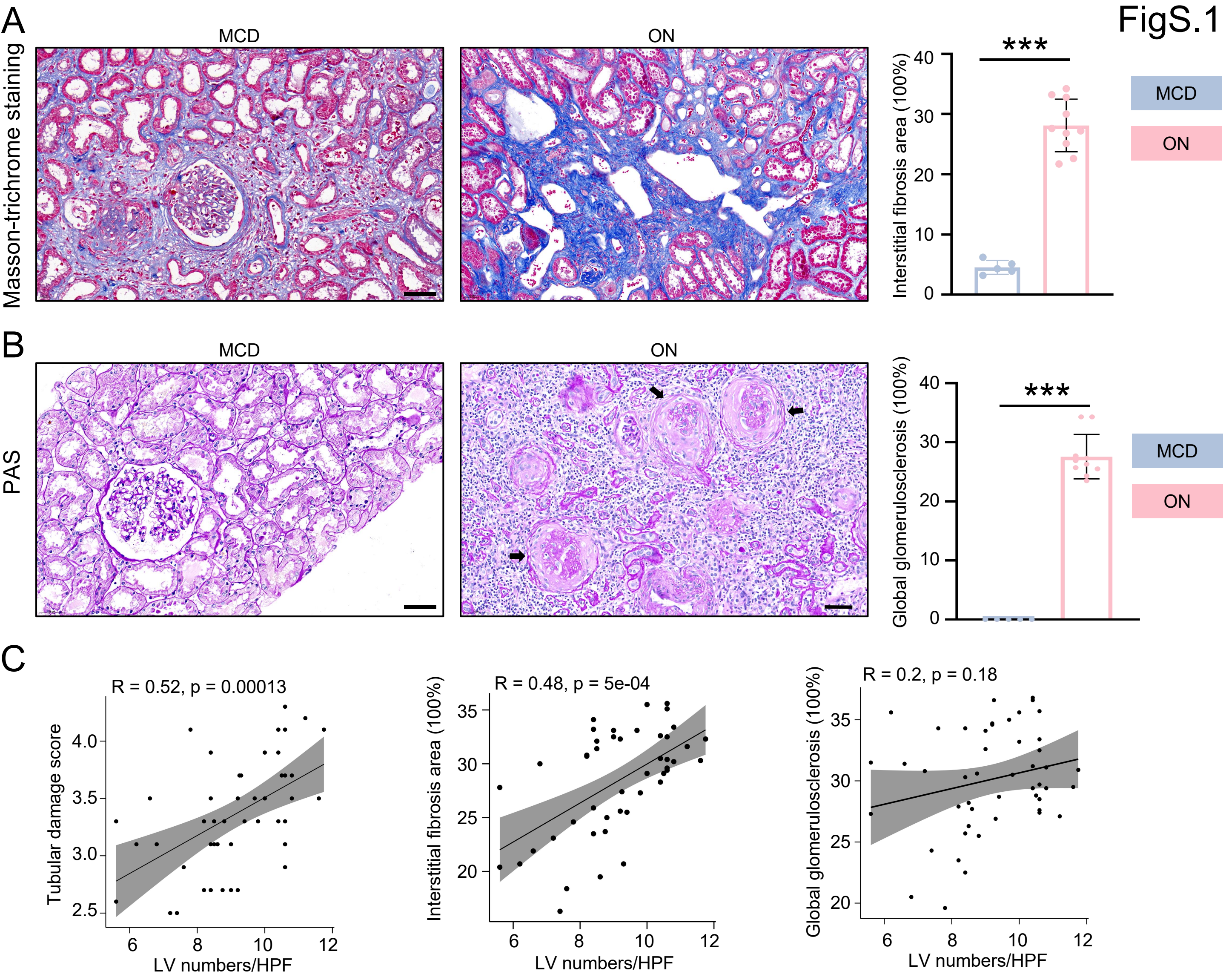
FigS.1
A
Masson-trichrome staining
MCD
ON
Interstitial fibrosis area (100%)
***
MCD
ON
B
PAS
MCD
ON
Global glomerulosclerosis (100%)
***
MCD
ON
C
R = 0.52, p = 0.00013
Tubular damage score
LV numbers/HPF
R = 0.48, p = 5e-04
Interstitial fibrosis area (100%)
LV numbers/HPF
R = 0.2, p = 0.18
Global glomerulosclerosis (100%)
LV numbers/HPF

Supp.Table 1. Histopathological features of enrolled patients with renal biopsy samples.

Histopathological features	Overall, N=48	LVs^{low}, N=22	LVs^{high}, N=26	p-value[1]
Interstitial fibrosis area	30.1 (25.4 – 32.3)	25.5 (22.2 – 31.3)	30.4 (29.1 – 32.5)	**0.023**
Tubular damage score	3.3 (3.1 – 3.7)	3.1 (2.7 – 3.3)	3.6 (3.3 – 3.9)	**< 0.001**
Global glomerulosclerosis proportion	30.4 (27.6 – 31.5)	28.1 (25.6 – 31.5)	31.0 (29.0 – 34.7)	**0.017**

The bold values indicate P < 0.05. Data are presented as median (25–75th percentiles). [1] Wilcoxon rank sum test. Based on the average lymphatic vessel density, the samples were categorized into high-density and low-density groups. LVs, lymphatic vessels.
